# Enhanced Influenza Vaccines Extend A(H3N2) Antibody Reactivity in Older Adults but Prior Vaccination Effects Persist

**DOI:** 10.1093/cid/ciaf060

**Published:** 2025-04-03

**Authors:** Annette Fox, Stephany Sánchez-Ovando, Louise Carolan, A Jessica Hadiprodjo, Yuyun Chen, Faith Ho, Samuel M S Cheng, Mark G Thompson, A Danielle Iuliano, Min Z Levine, Sophie A Valkenburg, Dennis K M Ip, J S Malik Peiris, Sheena G Sullivan, Benjamin J Cowling, Nancy H L Leung

**Affiliations:** Department of Infectious Diseases, University of Melbourne, at the Peter Doherty Institute for Infection and Immunity, Melbourne, Australia; Victorian Infectious Diseases Reference Laboratories, at the Peter Doherty Institute for Infection and Immunity, Melbourne, Australia; Department of Infectious Diseases, University of Melbourne, at the Peter Doherty Institute for Infection and Immunity, Melbourne, Australia; Victorian Infectious Diseases Reference Laboratories, at the Peter Doherty Institute for Infection and Immunity, Melbourne, Australia; Victorian Infectious Diseases Reference Laboratories, at the Peter Doherty Institute for Infection and Immunity, Melbourne, Australia; Department of Infectious Diseases, University of Melbourne, at the Peter Doherty Institute for Infection and Immunity, Melbourne, Australia; Victorian Infectious Diseases Reference Laboratories, at the Peter Doherty Institute for Infection and Immunity, Melbourne, Australia; School of Public Health, Li Ka Shing Faculty of Medicine, The University of Hong Kong, Hong Kong Special Administrative Region, China; School of Public Health, Li Ka Shing Faculty of Medicine, The University of Hong Kong, Hong Kong Special Administrative Region, China; School of Public Health, Li Ka Shing Faculty of Medicine, The University of Hong Kong, Hong Kong Special Administrative Region, China; Centers for Disease Control and Prevention, National Center for Immunization and Respiratory Diseases, Influenza Division, Atlanta, Georgia, USA; Centers for Disease Control and Prevention, National Center for Immunization and Respiratory Diseases, Influenza Division, Atlanta, Georgia, USA; Centers for Disease Control and Prevention, National Center for Immunization and Respiratory Diseases, Influenza Division, Atlanta, Georgia, USA; HKU-Pasteur Research Pole, School of Public Health, Li Ka Shing Faculty of Medicine, The University of Hong Kong, Hong Kong Special Administrative Region, China; Department of Microbiology and Immunology, Peter Doherty Institute for Infection and Immunity, University of Melbourne, Melbourne, Australia; School of Public Health, Li Ka Shing Faculty of Medicine, The University of Hong Kong, Hong Kong Special Administrative Region, China; School of Public Health, Li Ka Shing Faculty of Medicine, The University of Hong Kong, Hong Kong Special Administrative Region, China; Centre for Immunology and Infection (C2i), Hong Kong Science and Technology Park, Hong Kong Special Administrative Region, China; Department of Infectious Diseases, University of Melbourne, at the Peter Doherty Institute for Infection and Immunity, Melbourne, Australia; Victorian Infectious Diseases Reference Laboratories, at the Peter Doherty Institute for Infection and Immunity, Melbourne, Australia; Department of Epidemiology, University of California, Los Angeles, USA; School of Public Health, Li Ka Shing Faculty of Medicine, The University of Hong Kong, Hong Kong Special Administrative Region, China; Laboratory of Data Discovery for Health (D24H), Hong Kong Science and Technology Park, Hong Kong Special Administrative Region, China; School of Public Health, Li Ka Shing Faculty of Medicine, The University of Hong Kong, Hong Kong Special Administrative Region, China; Laboratory of Data Discovery for Health (D24H), Hong Kong Science and Technology Park, Hong Kong Special Administrative Region, China

**Keywords:** influenza, vaccination, immunogenicity, public health, older adults

## Abstract

**Background:**

Influenza vaccine effectiveness can be reduced in older adults and among repeatedly vaccinated groups. Results from year 1 of “PIVOT,” a randomized trial among adults aged ≥65 years in Hong Kong, showed that adjuvanted (Adj), high-dose (HD), and recombinant hemagglutinin (rHA) vaccines induced greater antibody responses against vaccine viruses than standard-dose (SD) influenza vaccine. Here, we examine the breadth of A(H3N2)-reactive antibodies induced during the first 2 study years (2017/2018, 2018/2019), and compare participants who received influenza vaccination annually, or not at all, for 5 years preceding enrollment.

**Methods:**

14–20 PIVOT participants per vaccine and prior vaccination group (0/5 or 5/5 prior years) who provided sera on days 0, 30, and 182 in year 1 and days 0 and 30 in year 2 were assessed. Hemagglutination inhibition (HAI) antibody titers were measured against 30 viruses spanning 1968 to 2018.

**Results:**

In year 1, rHA and Adj but not HD vaccines induced titers ≥40 and titer rises ≥4-fold (seroconversion) against significantly more strains than SD vaccine among participants vaccinated 0/5 prior years. Only rHA and Adj vaccines induced titers ≥40 against post-vaccine strains. Antibody responses were poor among participants vaccinated 5/5 compared with 0/5 prior years and only rHA increased the breadth of seroconversion compared with the SD vaccine in this group. Antibody responses were weaker across groups in year 2.

**Conclusions:**

The results suggest that Adj and particularly rHA vaccines may improve the breadth of protection against A(H3N2) viruses but may not overcome attenuating effects of repeated vaccination in older adults.

**Clinical Trials Registration:**

NCT03330132

## Teaser

Given the constant antigenic evolution of influenza viruses, antibody breadth may better indicate the protection potential of vaccines. We found that enhanced influenza vaccines elicit sustained increases in the breadth of antibody reactivity against A(H3N2) viruses in older adults.

Influenza vaccines are central to efforts to minimize the burden of influenza, particularly among high-risk groups such as older adults. The most widely used vaccines contain 15 µg of hemagglutinin (HA) from egg-grown influenza A(H1N1), A(H3N2), B-Victoria, and B-Yamagata viruses, which have been inactivated and detergent treated. In recent years, multiple countries have licensed adjuvanted (Adj), high-dose (HD), and recombinant-HA (rHA) vaccines in for use in older adults because standard dose (SD) vaccine has demonstrated reduced effectiveness in this age group [1–3]. Adjuvanted and HD are “enhanced” compared to SD vaccines by the inclusion of a squalene-based adjuvant (MF59) or 4 times more (60 µg) of each HA, respectively. Recombinant-HA contains 45 µg each of 4 baculovirus-expressed recombinant HAs that are expected to be more representative of circulating viruses than HAs of egg-grown viruses [4, 5]. Prior studies show that Adj [6, 7], HD [8, 9], and rHA [10] vaccines are more immunogenic and effective than SD vaccine in older adults.

The World Health Organization recommends annual influenza vaccination due to frequent updates in the vaccine's formulation to align with the evolution of circulating viruses [11]. Influenza A(H3N2) viruses undergo more rapid antigenic drift than other influenza viruses, resulting in greater diversity and more frequent updates to the recommended vaccine virus [12]. Identification of candidate A(H3N2) vaccine viruses that induce antibodies with sufficient breadth to neutralize all circulating clades is challenging. Unsurprisingly, vaccine effectiveness is generally lower against A(H3N2) than against A(H1N1) or influenza B viruses [13], and both immunogenicity and effectiveness against A(H3N2) tend to be worse among older compared with younger adults vaccinated with SD vaccines [14]. Poorer responses among older adults may be attributable to immune senescence as well as repeated vaccination [15]. Numerous studies have reported lower vaccine effectiveness [16–19] and immunogenicity [20–24] against A(H3N2) with increasing years of prior influenza vaccination. These effects may have more impact among older adults because of their high vaccination uptake and higher risk of severe outcomes following A(H3N2) infection [14].

PIVOT refers to “Potent Influenza Vaccination strategies in Older adults—randomized immunogenicity Trial.” This double-blind trial commenced in 2017 and compares the immunogenicity of repeated annual vaccination with SD, Adj, HD, or rHA vaccines among adults aged 65–82 years who reside in Hong Kong [25]. Results from year 1 of the trial showed that antibody responses against vaccine strains were greater after vaccination with Adj, HD, and rHA compared with SD vaccine [25]. However, vaccine strain–reactive antibody titers explain only part of the protection conferred by influenza vaccines [26]. Given the constant antigenic evolution of influenza viruses, the breadth of antibodies induced by different vaccines may be a better indicator of their protection potential. Therefore, this study examined whether the A(H3N2) strain coverage of antibodies induced differed by vaccine type among a subset of PIVOT participants vaccinated 0 or 5 of 5 preceding years.

## METHODS

### Study Design

This study is a retrospective serological analysis of samples collected during the first 2 years of PIVOT [25]. Participants were followed for 4 years and received randomly assigned study vaccines annually shortly before each winter influenza season. Random allocation for each year was performed at enrollment in study year 1. Northern hemisphere 2017/2018 formulation vaccines were used, including FluQuadri (Sanofi) (SD), Fluzone High Dose (Sanofi) (HD), FLUAD (Seqirus) (Adj), and Flublok (Sanofi) (rHA) vaccine.

**Figure 1.**
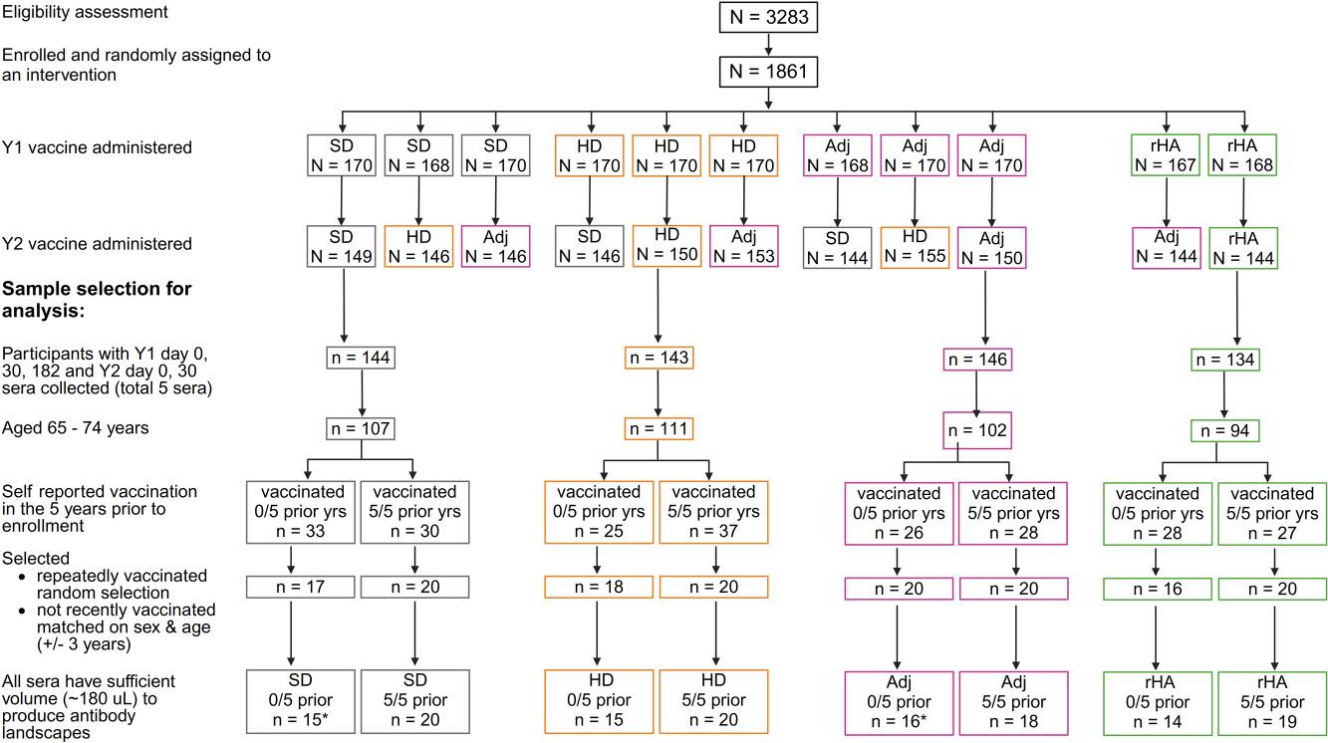
Participant enrollment in the PIVOT trial and sample selection for the current analysis. *Denotes that 3 participants in the 0/5 prior group (1 SD and 2 Adj recipients) were subsequently found to have prior vaccinations and were excluded from analysis. Abbreviations: Adj, adjuvanted; HD, high-dose; PIVOT, Potent Influenza Vaccination strategies in Older adults—randomized immunogenicity Trial; rHA, recombinant hemagglutinin; SD, standard-dose; Y1, year 1; Y2, year 2; yrs, years.

A subset of participants were assessed here (Figure 1), including those who (1) received the same study vaccine in study years 1 and 2; (2) were aged 65–74 years at enrollment (ie, born between 1942 and 1953); (3) self-reported receipt of influenza vaccination annually, or not at all, in the 5 years preceding enrolment (ie, vaccinated for 5/5 or 0/5 years between 2012/2013 and 2016/2017, respectively); and (4) provided sera for all 5 selected time points (year 1 day 0, 30 and 182 of vaccination and year 2 day 0 and 30 of vaccination). For each vaccine type, we first randomly selected 20 participants vaccinated 5/5 prior years, then selected 20 participants vaccinated 0/5 prior years by matching on sex and age (±3 years). Electronic (eHealth) records, accessed after participant selection, were used to identify vaccinations that were not self-reported. Self-reported vaccinations could not be validated because eHealth participation is voluntary.

All participants signed informed consent before any study-related procedures took place. The study protocol was approved by the Institutional Review Board of the University of Hong Kong.

### Viruses

Serum antibody titers were measured against 30 A(H3N2) viruses (Supplementary Table 1). Strains selected included prevailing and prior-year vaccine strains grown in embryonated hens’ eggs. We also included viruses representative of strains circulating between 1968 and 2018, grown in Madin-Darby canine kidney (MDCK) cells or MDCK cells stably transfected with α-2,6 sialyltransferase (Siat). Multiple cell-grown viruses circulating since 1999 were plaque selected on Siat cells to eliminate neuraminidase mutations that arose during prior growth in MDCK cells.

### Hemagglutination Inhibition Antibody Assay

Sera were treated with receptor-destroying enzyme (RDE; Denka Seiken), adsorbed with guinea pig red blood cells to remove nonspecific inhibitors, then serially diluted 2-fold, ranging from 1:10 to 1:10 240. Viruses were diluted to 4 hemagglutination (HA) units. Assays were performed using guinea pig red blood cells, as previously described [27]. Hemagglutination inhibition (HAI) titers were read using an automated hemagglutination analyzer (CypherOne; InDevR).

### Data Analysis

The HAI antibody titers and titer-fold rises were log_2_ transformed for all analyses. Results for individual viruses are presented as geometric mean titers (GMTs) and geometric mean fold-rises (GMRs) with 95% confidence intervals (CIs). Titers and fold-rises were also averaged across viruses grouped by circulation periods (2011–2013 and 2014–2018) and presented as GMTs and GMRs for each period. Generalized additive models (GAMs) were used to fit titer and titer-fold rise landscapes against viruses arranged temporally from 1968 to 2018 [13]. A random-effects term was included to account for variation between individuals and models were also run with adjustment for pre-vaccination titer. Titers and titer rises for viruses grouped by year of circulation were predicted from GAMs with 95% prediction intervals and plotted using geom ribbon. Antibody titer rises of 4-fold or greater were classified as seroconversion. The breadth of antibody responses was further examined by calculating the percentages of viruses against which each participant had titers of 40 or greater or seroconversion. GMTs, GMRs, and average percentages of viruses with titers of 40 or greater or seroconversion were compared using Wilcoxon rank-sum test with correction for comparison of multiple groups and time points (day 0, 30, 182, 365). Within each prior vaccination group, GMTs and GMRs were compared between each of the enhanced vaccines (HD, Adj, rHA) and the SD vaccine. For each vaccine type, GMTs and GMRs were also compared between groups vaccinated 0/5 versus 5/5 prior years. All analyses were performed in R version 4.2.3 (R Foundation for Statistical Computing, Vienna, Austria) using *tidyverse*, *mgcv*, *gtsummary*, and *rstatix* packages.

## RESULTS

### Participant Characteristics

Two hundred and thirty-four PIVOT participants met the selection criteria, of whom 151 (69%) were selected. Subsequently, 17 participants were excluded from analysis; 14 because serum volumes were insufficient to complete testing (Figure 1) and 3 because they reported being unvaccinated during 5 preceding years, but electronic records showed prior vaccinations. Table 1 shows characteristics of the 134 participants assessed, classified by vaccine received (SD, HD, Adj, and rHA) and vaccination history (received SD vaccine 0/5 vs 5/5 preceding years). The median age was between 67 and 70 years across groups. Proportions of male participants and with a medical condition were not statistically significantly different. None had a detectable influenza A infection during the period of this sub-study.

**Table 1.** Participant Characteristics by Vaccine Type and Years Vaccinated Prior to Enrollment

	SD		HD		Adj		rHA		*P*
	0/5 Prior	5/5 Prior	0/5 Prior	5/5 Prior	0/5 Prior	5/5 Prior	0/5 Prior	5/5 Prior	
No.	14	20	15	20	14	18	14	19	
Age, median (IQR), y	68 (66, 72)	69 (68, 70)	67 (66, 68)	69 (67, 70)	68 (66, 70)	68 (67, 71)	68 (66, 70)	70 (69, 72)	.053^a^
Male, n (%)	6 (43%)	7 (35%)	4 (27%)	5 (25%)	10 (71%)	10 (56%)	7 (50%)	6 (32%)	.12^b^
Medical condition,^c^ n (%)	9 (64%)	15 (75%)	8 (53%)	14 (70%)	11 (69%)	12 (67%)	11 (79%)	16 (84%)	.70^b^

Abbreviations: Adj, adjuvanted; COPD, chronic obstructive pulmonary disease; HD, high-dose; IQR, interquartile range; rHA, recombinant hemagglutinin; SD, standard-dose.

^a^Kruskal–Wallis test comparing all groups; comparisons between SD and other vaccines within each prior vaccination group were not significant; comparisons by prior vaccination status within each vaccine type were not significant except for rHA (*P* = .026).

^b^Fisher's exact test comparing all groups; comparisons between SD and other vaccines within each prior vaccination group were not significant; comparisons by prior vaccination status within each vaccine type were not significant.

^c^Any self-reported pre-existing medical conditions including heart problems (eg, coronary heart disease), hypertension, stroke, chronic lung diseases (eg, asthma and COPD), kidney diseases, liver diseases (eg, chronic hepatitis), cancers, diabetes, neurologic disorders (eg, epilepsy and Parkinson's disease), osteoarthritis, autoimmune diseases (eg, lupus and rheumatoid arthritis), and other health problems not listed.

### Effect of Vaccine Type and Prior Vaccination on the Breadth of A(H3N2) Antibodies

**Figure 2.**
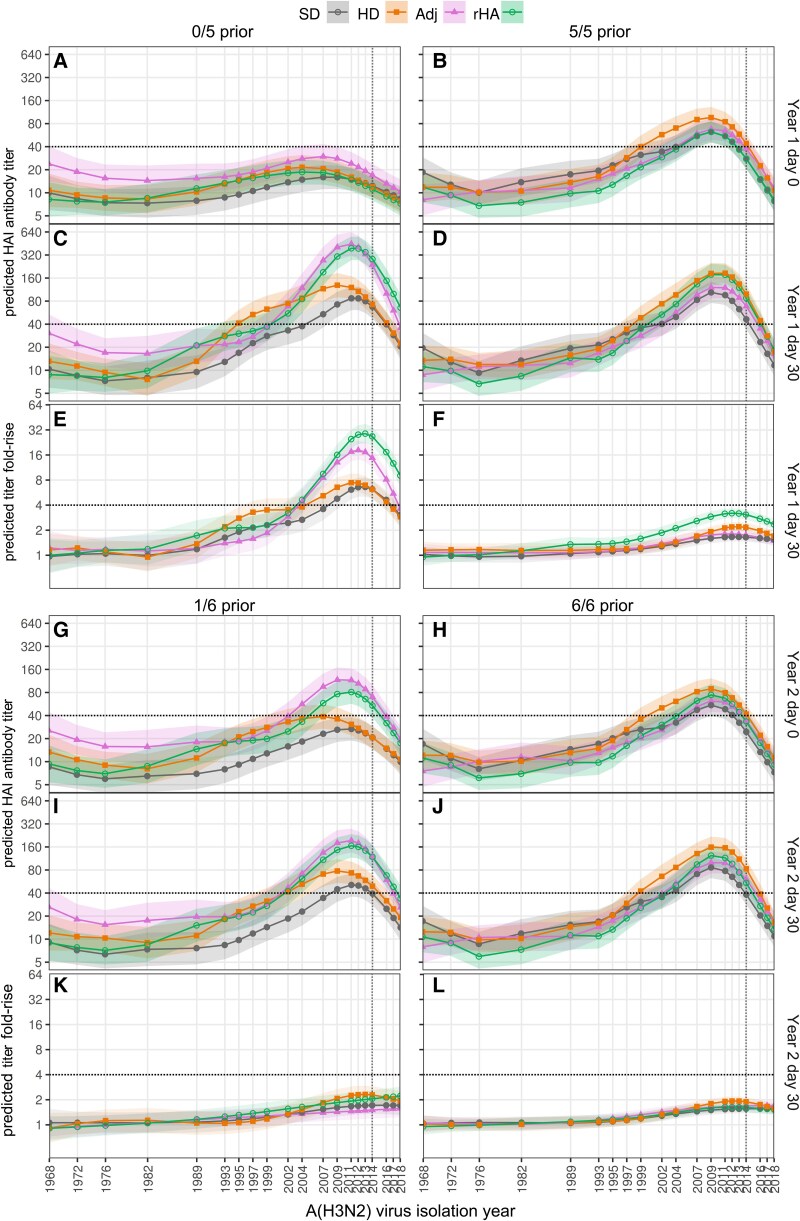
Antibody titer and titer-rise landscapes across A(H3N2) viruses vary by prior vaccination status and vaccine type. Generalized additive models (GAMs) were used to fit HAI antibody titers (*A–D*, *G–J*) and titer rises (*E–F*, *K–L*) against virus isolation year for 30 A(H3N2) viruses spanning 1968–2018. Symbols represent predicted values from GAMs for each virus year, time point, and vaccine type (see legend). Shading indicates 95% prediction intervals. The vertical dashed line indicates the year of the prevailing vaccine strain (HK14 or Si16). Abbreviations: Adj, adjuvanted; HAI, hemagglutination inhibition; HD, high-dose; rHA, recombinant hemagglutinin; SD, standard-dose.

At study commencement (year 1, day 0), titers were significantly higher against viruses spanning 1995 to 2018 among participants vaccinated 5/5 compared to 0/5 prior years, whereas titers against older strains were similar (Figure 2*A* and 2*B*). Vaccination in year 1 induced titer rises exceeding 4-fold (seroconversion) against viruses spanning 2007–2018 and substantial change to antibody landscapes among participants vaccinated 0/5 but not 5/5 prior years (Figure 2*C*–*F*). Similar trends were observed when data were presented as raw titers for each vaccine and prior vaccination combination and as GMTs and GMRs for each virus (Supplementary Figures 1–3, Table 1).

All participants were born before the 1968 A(H3N2) pandemic, but titers were higher against contemporary compared with 1968 ancestral-type strains (Figure 2*A* and *B*; Supplementary Figure 2*A*), and vaccination induced negligible titer rise against ancestral strains (Figure 2*E* and 2*F*, Supplementary Figure 2*B*).

Among participants vaccinated 0/5 prior years, Adj and rHA vaccines induced substantially greater antibody titers and titer rises than SD vaccine (Figure 2*C* and 2*E*). Post-vaccination titers were higher across strains spanning 2009–2017 for Adj and rHA compared with SD vaccine recipients (Figure 2*C*, Supplementary Figure 3*B*). On average, Adj, rHA, and SD vaccinees had post-vaccination titers of 40 or greater against 89%, 87%, and 65% of viruses circulating since 2007, respectively, with consistent trends 182 days after vaccination (Table 2). Similarly, titer fold-rises were greater among Adj and rHA compared with SD vaccine recipients (Figure 2*E*, Supplementary Figure 3*C*) who seroconverted against 83%, 83%, and 59% of viruses circulating since 2007, respectively (Table 2). A time course of antibody responses against the A(H3N2) vaccine strain (A/Hong Kong/4801/2014) indicates that titers remained significantly higher among Adj and rHA compared with SD vaccine recipients for up to 1 year after vaccination (Supplementary Figure 4). Although rHA vaccine is more representative of cell-grown than egg-grown virus, there was no indication that rHA induced antibodies that were more oriented towards cell-grown over egg-grown viruses when compared with SD vaccine (Supplementary Figure 5). The HD vaccinees had higher post-vaccination titers against some strains than SD vaccinees (Figure 2*C*, Supplementary Figure 3*B*), but titer rises were not significantly different (Figure 2*E*, Supplementary Figure 3*C*).

**Table 2.** Average Percentages of Viruses Recognized From 2007 Forward by Vaccine Type and Prior Vaccination Status

Vaccine	0/5 Prior Years Vaccinated		5/5 Prior Years Vaccinated		0/5 vs 5/5 Prior
	Mean (95% CI)	*P* ^a^	Mean (95% CI)	*P*	*P* ^a^
Percentage of viruses with titers ≥40^b^					
Day 0					
SD	21 (3–38)	Reference	44 (34–54)	Reference	.007
HD	19 (6–31)	1	56 (46–66)	.342	<.001
Adj	24 (10–38)	.366	51 (37–65)	.964	.016
rHA	16 (7–26)	1	40 (28–51)	.964	.008
Day 30					
SD	65 (47–82)	Reference	57 (47–66)	Reference	.252
HD	65 (49–81)	.817	75 (69–81)	.019	.801
Adj	89 (85–94)	.059	66 (54–77)	.212	.005
rHA	87 (76–98)	.059	71 (60–82)	.076	.008
Day 182					
SD	47 (30–64)	Reference	53 (44–62)	Reference	.769
HD	49 (35–63)	1	67 (59–75)	.122	.051
Adj	73 (62–85)	.080	57 (44–71)	1	.17
rHA	74 (61–87)	.080	56 (43–69)	1	.049
Percentage of viruses against which participants seroconverted^c^					
Day 30					
SD	59 (39–80)	Reference	15 (2–28)	Reference	.002
HD	64 (48–80)	.982	25 (11–38)	.123	.002
Adj	83 (69–97)	.306	16 (6–26)	.151	<.001
rHA	83 (70–97)	.291	49 (33–64)	.006	.001
Day 182					
SD	38 (20–56)	Reference	6 (0–11)	Reference	<.001
HD	36 (20–51)	.660	7 (0–13)	1	.002
Adj	57 (40–74)	1	4 (0–9)	1	<.001
rHA	72 (56–87)	.066	17 (5–29)	.280	<.001

Abbreviations: Adj, adjuvanted; CI, confidence interval; HD, high-dose; rHA, recombinant hemagglutinin; SD, standard-dose.

^a^ *P* values are for 2-sided Wilcoxon test with Holm correction for multiple comparisons.

^b^For each participant and time point, and for 18 viruses spanning 2007–2018, the percentage of viruses against which titers were ≥40 was calculated, then used to calculate mean percentages for each vaccine and prior vaccination group.

^c^As in footnote “b”, the percentage of viruses against which titers rose ≥4-fold was calculated, then used to calculate mean percentages for each vaccine and prior vaccination group.

For participants vaccinated 5/5 prior years, only rHA vaccine induced greater post-vaccination titers and titer rises than SD vaccine (Figure 2*D* and *F*, Supplementary Figures 3*B* and *C*). However, by day 182, there was no clear advantage of rHA in terms of breadth of titers of 40 or greater or seroconversion (Table 2). One year after vaccination, antibody titers remained higher than pre-vaccination levels among participants vaccinated 0/5 prior years who received enhanced vaccines, whereas titer landscapes were similar to baseline among those who received SD vaccine or who were vaccinated 5/5 prior years (Supplementary Figure 6). Vaccination in year 2 induced weaker titer fold-rises across viruses that did not differ significantly between vaccines or prior vaccination groups (Figure 2*K* and *L*).

These results indicate that enhanced vaccines provide sustained increases in the breadth of immunity induced against A(H3N2) viruses, particularly when administered in the absence of prior vaccination, with the greatest increases provided by rHA followed by Adj vaccine.

### Induction of Antibodies Against Strains Circulating Before or After the Vaccine Strain

**Figure 3.**
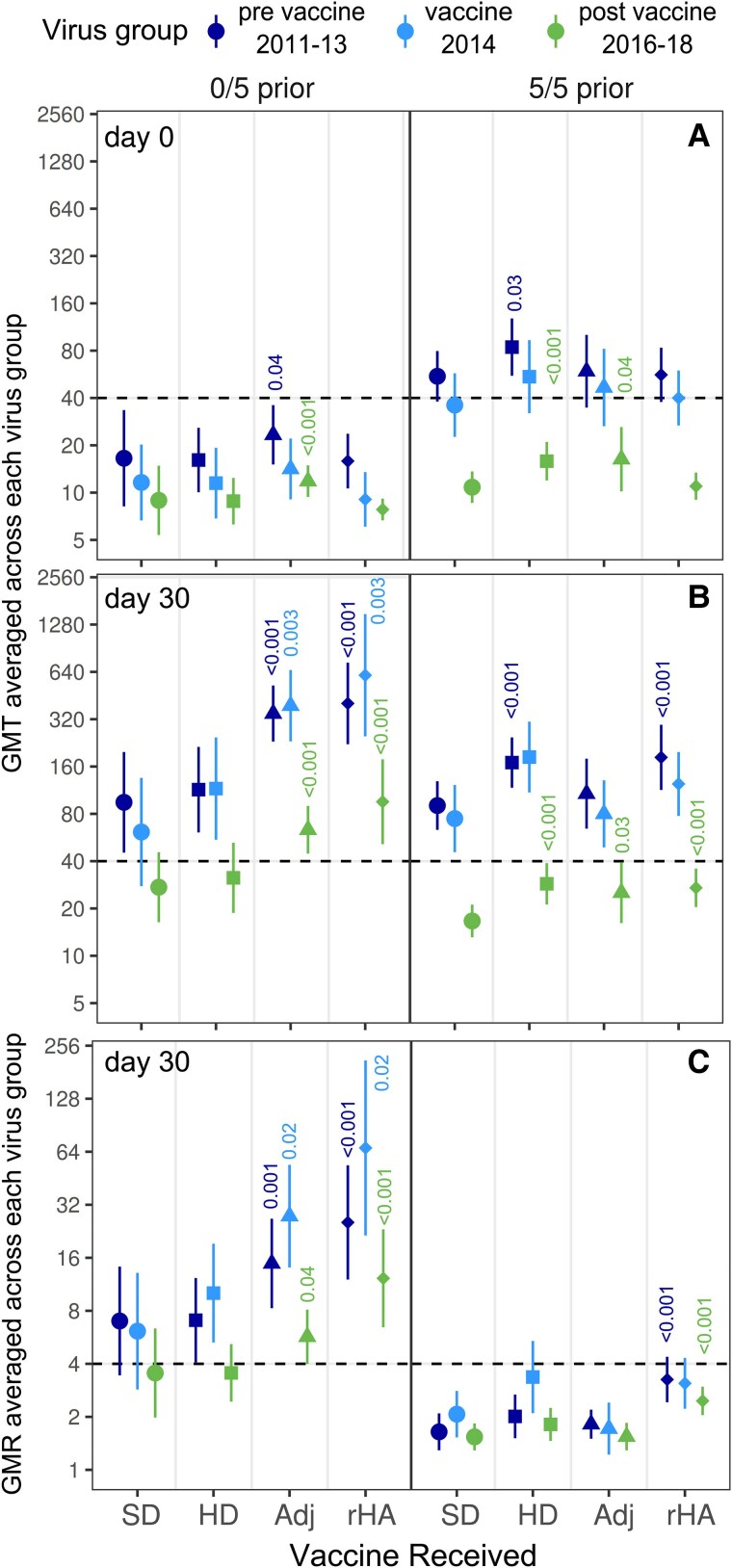
HAI antibody titers and titer rises averaged across A(H3N2) viruses grouped by year of circulation. Geometric means are shown for antibody titers and fold-rises averaged across A(H3N2) viruses spanning 2011–2013 (pre-vaccine: dark blue) or 2016–2018 (post-vaccine: green), and for the vaccine strain (HK14e: light blue) in study year 1 (2017/2018). Participants are grouped by vaccine received and number of prior years vaccinated. (*A*, *B*) GMTs on days 0 and 30. (*C*) GMRs on days 30. Error bars represent 95% CIs. Values above error bars are *P* values < .05 for 2-sided Wilcoxon tests comparing enhanced vaccines with SD vaccine by virus group and time point. Abbreviations: Adj, adjuvanted; CIs, confidence intervals; GMR, geometric mean fold-rise; GMT, geometric mean titer; HAI, hemagglutination inhibition; HD, high-dose; rHA, recombinant hemagglutinin; SD, standard-dose.

To further examine whether the focus of antibodies differs by vaccine type and prior vaccination, titers and titer rises were averaged across viruses circulating before and after A/Hong Kong/4801/2014, the vaccine strain in year 1. On day 0, all groups had higher titers against strains circulating before the vaccine strain than against the vaccine strain and lowest titers against post-vaccine strains (Figure 3*A*). This trend persisted after vaccination for participants who received SD vaccine or who had been vaccinated 5/5 prior years. However, titers and fold-rises were at least as high against the vaccine strain as against pre-vaccine strains among participants vaccinated 0/5 prior years who received enhanced vaccines (Figure 3*B* and 3*C*). Adjuvanted and rHA vaccines were the only vaccines that induced titers of 40 or greater and seroconversion against post-vaccine strains (Figure 3*B* and 3*C*). On average, titers were 40 or greater against 74% of post-vaccine strains among Adj and rHA vaccine recipients who were vaccinated 0/5 prior years compared to 40% and 47%, respectively, for those vaccinated 5/5 prior years. These results suggest that Adj and rHA vaccines may be more effective than SD vaccine at updating immunity towards prevailing and future strains.

### Effects of Vaccine Type and Prior Vaccination After Adjusting for Pre-vaccination Titer

**Figure 4.**
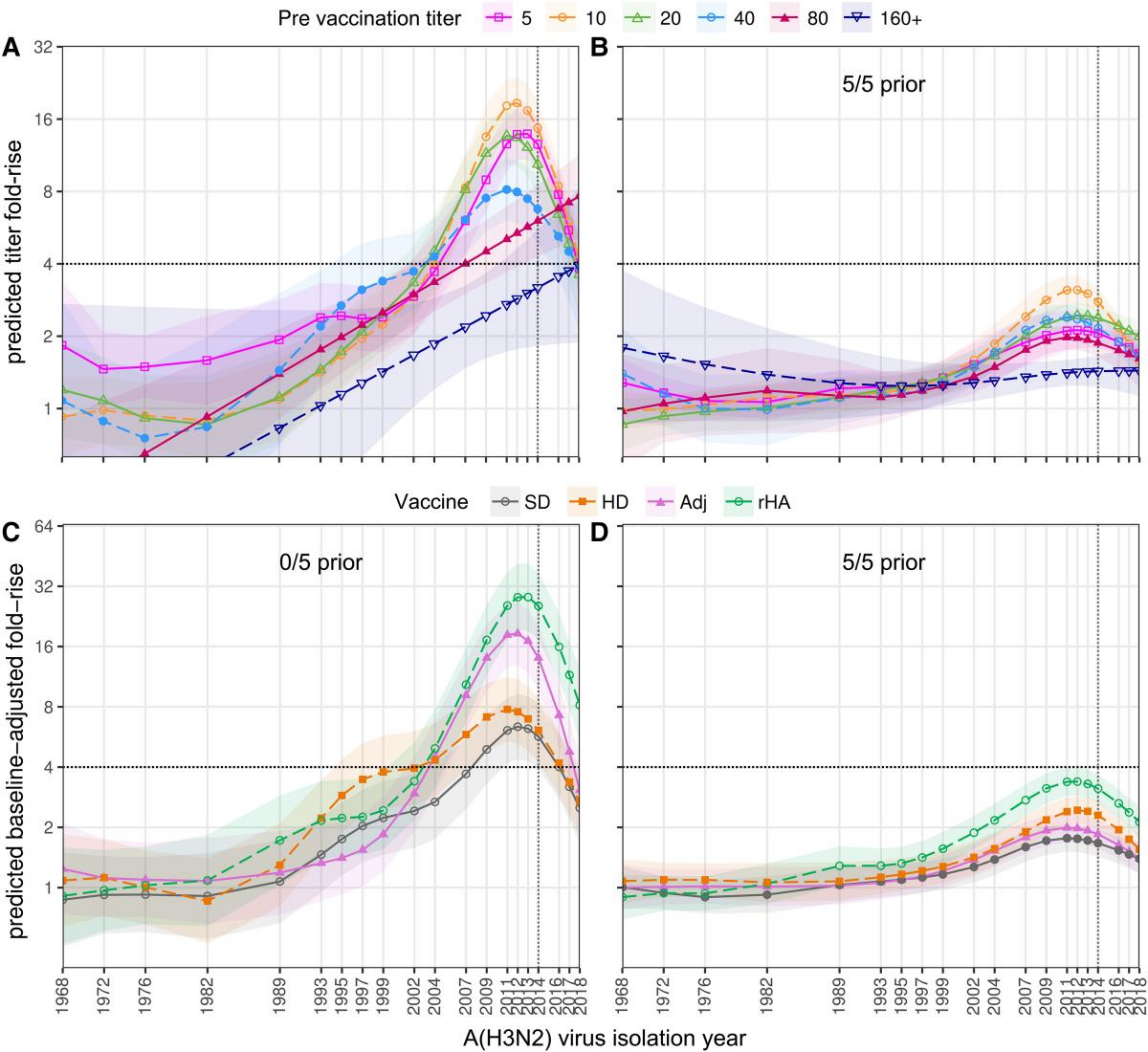
Effect of pre-vaccination HAI antibody titer on titer fold-rise against A(H3N2) viruses. Generalized additive models (GAMs) were used to fit HAI antibody titer fold-rises against virus isolation year (1968–2018). (*A*, *B*) Data were analyzed separately for participants who had been vaccinated 0/5 or 5/5 prior years and stratified by pre-vaccination titer (legend above plots). (*C*, *D*) GAMs adjusted for pre-vaccination titer. Data were analyzed separately for participants who had been vaccinated 0/5 or 5/5 prior years and stratified by vaccine received (legend above plots). Results are shown for year 1. Dashed vertical lines indicate the year of the prevailing vaccine strain (HK14). Dashed horizontal lines indicate the threshold for seroconversion. Abbreviations: Adj, adjuvanted; HAI, hemagglutination inhibition; HD, high-dose; rHA, recombinant hemagglutinin; SD, standard-dose.

Previous studies show that antibody titer rise can decline with increasing pre-vaccination titer [28, 29]. This was also apparent in the current study among participants vaccinated 0/5 prior years where titer rise against vaccine-proximal viruses decreased as pre-vaccination titers increased from 20 through 320+ (Figure 4*A*). For each level of pre-vaccination titer, fold-rises across vaccine proximal strains were lower among participants vaccinated 5/5 prior years (Figure 4*B*) compared to 0/5 prior years (Figure 4*A*). Similarly, predicted titer rises after adjusting for pre-vaccination titer were higher among participants who had been vaccinated 0/5 compared to 5/5 prior years (Figure 4*C* and 4*D*), indicating that factors other than pre-existing antibodies contribute to attenuating effects of repeated prior vaccination. Adjusted titer rises were highest among participants vaccinated 0/5 prior years who received rHA vaccines, followed by those who received Adj vaccine, as per unadjusted analysis.

## DISCUSSION

This study examined whether enhanced influenza vaccines extend the breadth of antibodies induced against A(H3N2) viruses and whether they overcome the attenuation that has been associated with repeated administration of SD vaccine. We found that Adj and rHA vaccines were substantially more immunogenic than SD vaccine in older adults who had not been vaccinated during 5 prior years. They induced larger antibody fold-rises, yielding seroconversion and titers of 40 or greater against a broader range of strains. Adjuvanted and rHA were also the only vaccines studied that induced seroconversion and titers of 40 or greater against subsequently circulating viruses. Although rHA vaccine also showed improved immunogenicity among participants vaccinated 5/5 prior years, immunogenicity was substantially attenuated compared with those vaccinated 0/5 prior years. Importantly, enhanced vaccines durably shifted antibody landscapes forward, so that titers against 2009–2018 viruses were higher 1 year after vaccination than before vaccination, but only among those who were not recently vaccinated.

The strong attenuating effect of prior vaccination on antibody responses was observed for all vaccines studied and was indicated by both the comparison of participants vaccinated 0/5 versus 5/5 prior years and of responses to vaccination in year 1 versus year 2. Vaccination was particularly poor at inducing titers of 40 or greater against post-vaccine strains among participants vaccinated 5/5 prior years, which could impact protection. Our results are consistent with a recent report that PIVOT study participants vaccinated in the prior (2016/2017) year had lower GMTs and GMRs against HK14 than those who were not vaccinated [30]. Similarly, a 2019–2020 vaccine trial involving adult healthcare professionals found that rHA immunogenicity against A(H3N2) declined when given for a second consecutive year [31]. It has been suggested that pre-existing antibodies may mask or clear antigen so that responses to vaccination are attenuated among previously vaccinated individuals [32]. While our analysis, showing that antibody titer rises decrease with increasing pre-vaccination titer, supports the concept of antibody masking, it also suggests that factors other than pre-existing antibody contribute to the attenuating effects of prior vaccination. A study using surface plasmon resonance off-rates as a measure of antibody affinity for HA1 subunit proteins indicated a sharp, but short-lived, rise in affinity maturation after first-time vaccination, but substantially less affinity maturation after repeat vaccination [33]. The authors suggest that this may indicate that high-affinity antibodies are primarily generated by short-lived plasmablasts, and that the source of these plasmablasts is not replenished [33].

Antibody landscapes were not characteristic of original antigenic sin or seniority whereby strains encountered earlier in life are higher in the antibody hierarchy [34]. We have reported this previously for adults born before 1968, whereas antibody titers are highest against early-life strains for younger adults [27]. Antigenic sin/seniority hypotheses suggest that contemporary virus exposures recall memory against epitopes conserved with earlier strains [27]. However, titer rise was negligible against ancestral strains, indicating that there is limited HAI antibody epitope conservation between 1968 and 2014 viruses.

Recombinant HA induced the greatest and broadest antibody titer rises followed by Adj vaccine, whereas HD vaccine was only marginally better than SD vaccine. These findings are somewhat consistent with a vaccine-effectiveness study that found that rHA is moderately more effective than the other 3 vaccines, while HD and Adj are more effective than SD vaccine [35]. Most other studies comparing vaccine platforms assessed antibodies against vaccine antigens only. These studies generally indicated that rHA is more immunogenic against A(H3N2) than HD and/or Adj vaccines, and that HD and Adj are both more immunogenic than SD vaccines, whereas relationships between Adj and HD vaccines vary between studies [31, 36–38]. Recombinant HA vaccines contain 45 µg of each rHA representative of circulating virus sequences, whereas HD vaccines contain 60 µg of each HA extracted from inactivated egg-grown virus. Although A(H3N2) viruses change antigenically when grown in eggs, rHA induced greater antibody responses against both cell and egg viruses. In contrast, another recent study found that rHA significantly reduced the ratio of antibody binding to egg-grown over cell-grown A(H3N2) virus [39]. We previously found that rHA vaccine induced a greater rise in HA-reactive CD4 T cells than other vaccines [25]. One possibility may be that rHA is more immunogenic than HD vaccine because the protein is in a more native state than egg-grown vaccine antigen, which has been treated with detergents and inactivation reagents.

The main limitations of our study are that the sample sizes per vaccine and prior vaccination group were small, demographic characteristics were not uniform at baseline, and vaccination histories could not be validated. These are unlikely to affect the inference that rHA induces broader immunity since differences in landscapes were substantial, but may account for differences in effects of HD vaccines in the current compared with previous studies. We do not know whether participants had recent influenza infections, which can be associated with improved vaccine immunogenicity [27].

In summary, this study indicates that enhanced vaccines, most particularly rHA vaccine but also Adj vaccine, may substantially improve the breadth of protection induced against influenza A(H3N2) viruses in older adults. Additional gain in immunogenicity by enhanced vaccines (including Adj and rHA) compared with SD in the first year of recent vaccinations may be subsequently offset by repeated vaccinations in the following years. It will be important to elucidate the mechanisms underlying attenuation, and to understand whether rHA and Adj vaccines augment immunogenicity via similar or discrete mechanisms that may be complementary.

## Supplementary Material

ciaf060_Supplementary_Data

## Supplementary Data

Supplementary materials are available at *Clinical Infectious Diseases* online. Consisting of data provided by the authors to benefit the reader, the posted materials are not copyedited and are the sole responsibility of the authors, so questions or comments should be addressed to the corresponding author.

## Notes

***Author contributions.*** A. F., N. H. H. L., S. G. S., and B. J. C. designed and led this sub-study. B. J. C. and M. G. T. designed the PIVOT trial. S. S.-O., L. C., and A. J. H. performed serology. A. F. cleaned and analyzed data and drafted the manuscript. N. H. H. L. and B. J. C. contributed to drafting the manuscript. Y. C., S. M. S. C., F. H., and D. K. M. I. oversaw participant investigation, sample processing, data management, and clinical oversight. A. D. I., M. Z. L., S. A. V., J. S. M. P., and S. G. S. contributed to data interpretation. All authors revised the paper.

***Acknowledgments.*** The authors thank the participants. They also thank the study staff for assistance in project implementation, including Tin-Kin Chau, Jennifer Ho, Fiona Kee, Janisy Lai, Cecily Leung, Anita Li, Jessamine Luk, Loretta Mak, Yvonne Ng, Teresa So, Angel Wong, Miyuki Wong, Phoebe Wong, and Kitty Yu; Leo Luk, Emily Yau, Chi Tsang, and Kin Chan for laboratory support; and Julie Au and Chi-Kin Lam for administrative support.

***Financial support.*** This work was supported by the US Centers for Disease Control and Prevention (cooperative agreement number IP001064-02 to B. J. C.), the National Institute of Allergy and Infectious Diseases (grant number R01 AI170116 to B. J. C.), and the Theme-based Research Scheme (project number T11-712/19-N to B. J. C.) of the Research Grants Council (RGC) of the Hong Kong Special Administrative Region, China. B. J. C. is also supported by an RGC Senior Research Fellowship from the University Grants Committee of Hong Kong (grant number: HKU SRFS2021-7S03). N. H. L. L. is supported by a Health and Medical Research Fund Research Fellowship Scheme (grant number 07210157) from the Health Bureau of the Hong Kong Special Administrative Region, China. The Collaborating Centre for Reference and Research on Influenza is funded by the Australian Government Department of Health.
