# Supplementary material for: Enhanced Influenza Vaccines Extend A(H3N2) Antibody Reactivity in Older Adults but Prior Vaccination Effects Persist
Source: Clin Infect Dis. 2025 Apr 3;81(4):e192–201. doi: 10.1093/cid/ciaf060 (PMC12596410; doi:10.1093/cid/ciaf060)
Supplement: ciaf060_Supplementary_Data [file ciaf060_supplementary_data.docx]

**Table S1. A(H3N2) viruses assessed and Geometric Mean Titres (GMTs) in Year 1, comparing participants vaccinated 0/5 versus 5/5 prior years**

|  |  |  |  |  | **Day 0 GMTs** ^d^ | | **Day 30 GMTs** ^d^ | |
| --- | --- | --- | --- | --- | --- | --- | --- | --- |
| **Virus Designation** | **Abbreviation** | **Passage** ^a^ | **Accession** ^b^ | **Years in Vaccine** ^c^ | **0/5 prior** | **5/5 prior** | **0/5 prior** | **5/5 prior** |
| A/Bilthoven/16190/68 | Bi68 | X,M3 | EPI1868399 |  | 12.6 | 12.2 | 13.4 | 12.8 |
| A/Bilthoven/21793/72 | Bi72 | M3 | EPI1868401 |  | 11.2 | 10.9 | 12.9 | 11.9 |
| A/Bilthoven/1761/76 | Bi76 | M3 | EPI1868402 |  | 7.7 | 7.8 | 8.3 | 8.3 |
| A/Bilthoven/2271/76 | Bil76 | X,M3 | EPI1868403 |  | 9.1 | 10.3 | 10.4 | 10.5 |
| A/Philippines/2/82 | Ph82 | MX,M2 | EPI1868405 |  | 10.0 | 10.8 | 10.6 | 11.5 |
| A/Netherlands/620/89 | Ne89 | X,M1,M3 | EPI1868433 |  | 10.4 | 12.1 | 12.9 | 14.5 |
| A/Netherlands/179/93 | Ne93 | X, M3 | EPI1868435 |  | 13.2 | 16.4 | 27.1 | 19.3 |
| A/Netherlands/178/95 | Ne95 | 293T,M4 | EPI1868436 |  | 16.5 | 23.7 | 33.3 | 29.2 |
| A/Tasmania/1/97 | Ta97 | M7 | EPI1868440 |  | 8.0 | 10.4 | 15.9 | 12.0 |
| A/Townsville/2/1999 | Tv99 | S plaque, S1 |  |  | 23.1 | 50.5 | 65.1 | 65.0 |
| A/Philippines/472/02 | Ph02 | S plaque, S3 | EPI1868438 |  | 20.2 | 41.5 | 67.5 | 60.6 |
| A/Victoria/511/2004 | Vi04 | S plaque, S3 | EPI1868443 |  | 16.2 | 31.7 | 42.5 | 43.8 |
| A/Brisbane/10/2007 | Br07 | S plaque, S4 | EPI1868482 |  | 33.7 | 87.6 | 223.5 | 161.4 |
| A/Perth/16/2009 | Pe09 | S plaque,S2 | EPI1868486 |  | 22.6 | 80.7 | 249.8 | 173.5 |
| A/Perth/16/2009 | Pe09e | E7 | EPI1868485 | 2010/11, 2011/12 | 8.5 | 24.2 | 62.0 | 47.0 |
| A/Victoria/361/2011 | Vi11 | S plaque,S2 | EPI1868575 |  | 29.9 | 124.4 | 380.5 | 269.7 |
| A/Victoria/361/2011 | Vi11e | E6 | EPI1868550 | 2012/13 | 19.8 | 82.9 | 197.5 | 164.4 |
| A/Texas/50/2012 | Tx12 | S plaque,S4 | EPI1868581 |  | 31.0 | 125.5 | 487.4 | 295.1 |
| A/Texas/50/2012 | Tx12e | E5,E2 | EPI1868551 | 2013/14, 2014/15 | 19.3 | 87.5 | 249.8 | 183.1 |
| A/Switzerland/9715293/13 | Sw13 | S9 | EPI1868576 |  | 9.5 | 20.9 | 78.0 | 41.5 |
| A/Switzerland/9715293/13 | Sw13e | E7 | EPI1868552 | 2015/16 | 9.2 | 25.4 | 84.1 | 51.6 |
| A/New Caledonia/104/2014 | NC14 | S plaque, S7 | EPI1868582 |  | 11.3 | 20.0 | 106.3 | 45.0 |
| A/Hong Kong/4801/14 | HK14e | E8 | EPI1868553 | 2016/17, **2017/18** | 11.4 | 43.8 | 204.9 | 108.6 |
| A/Newcastle/30/2016 | Nc16 | S5 | EPI1868578 |  | 11.4 | 21.3 | 90.5 | 42.2 |
| A/Singapore/16-0019/16 | Si16e | E5+1+E1 | EPI1381189 | **2018/19** | 10.0 | 19.6 | 106.3 | 43.4 |
| A/Kansas/14/2017 | Ka17 | S4 | EPI1868579 |  | 7.7 | 8.2 | 22.9 | 12.4 |
| A/Kansas/14/2017 | Ka17e | E9 | EPI1318832 | 2019/20 | 7.7 | 10.4 | 21.8 | 16.9 |
| A/Brisbane/60/2018 | Br18 | S3 | EPI1868580 |  | 10.0 | 14.3 | 50 | 26.9 |
| A/Switzerland/8060/2017 | Sw17e | E7 | EPI1322581 |  | 10.5 | 18.6 | 121.9 | 39.6 |
| A/Sydney/22/2018 | Sy18 | S plaque, S5 | EPI1485365 |  | 7.8 | 7.4 | 20.2 | 10.7 |

a: passaged (+/- plaque selected) in embryonated hen’s eggs (E); Madin-Darby canine kidney (M) cell or MDCK cells stably transfected with α-2,6 sialyltransferase (S)

b: accession codes for HA gene sequences in GISAID, <https://gisaid.org/>.

c: Strains in Northern Hemisphere influenza vaccines. The five vaccine years preceding participant enrolment are underlined. Study years 1 and 2 are in bold

d: GMTs shaded in red differ by ≥ 2-fold between participants with 0/5 versus 5/5 prior vaccinations.


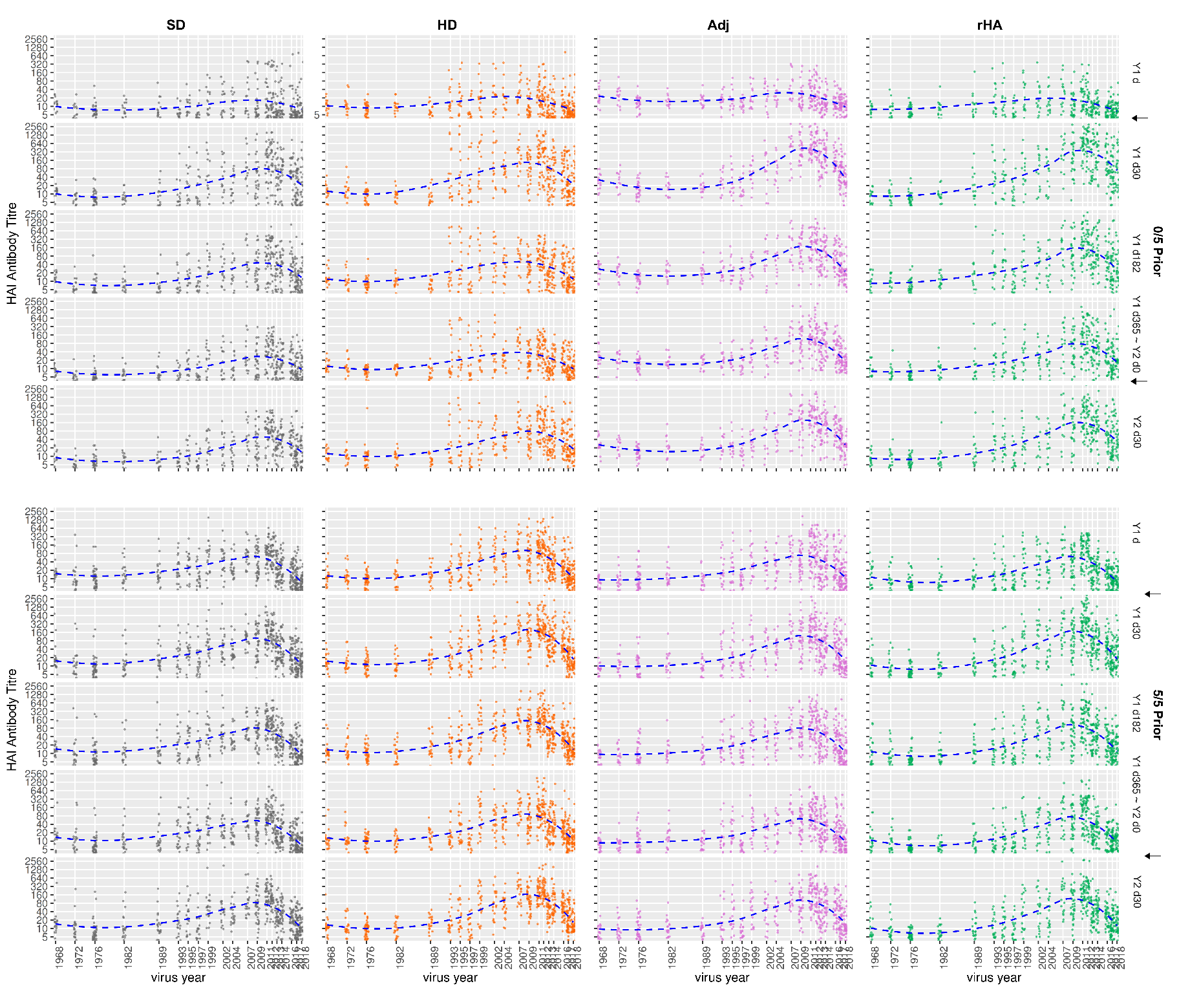


**Figure S1.** **Raw HAI antibody titres against A(H3N2) viruses spanning 1968 to 2018 by vaccine type, prior vaccination status and time-point.** Each dot represents a unique titre for each sample and virus combination. Samples were collected from each participant at five timepoints – days 0, 30 and 2812 in year 1 and days 0 and 30 in year 1. Arrows indicate the timing of vaccination. To aid visualization, Loess function was used to fit curves shown as dashed blue lines on each plot.


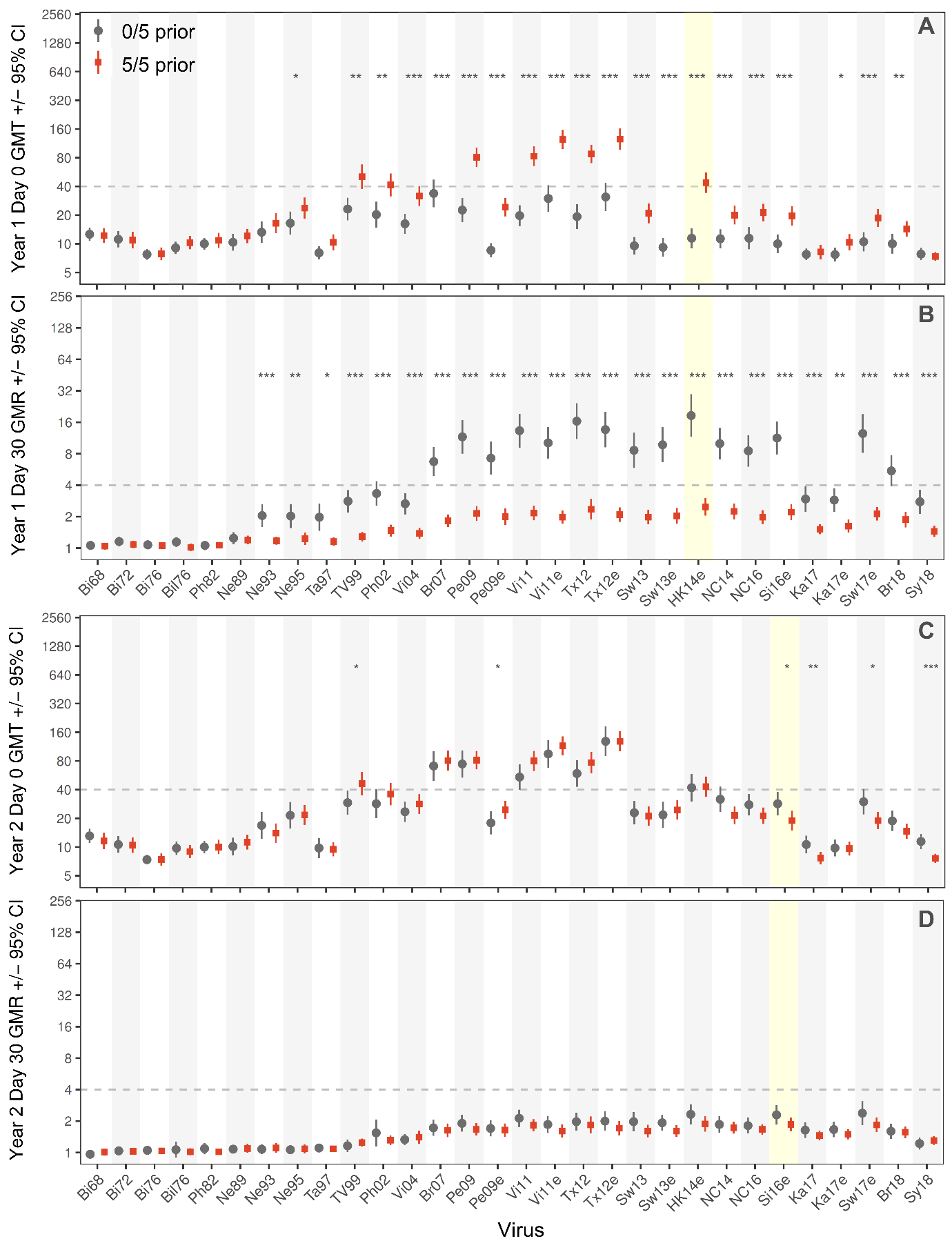


**Figure S2. HAI antibody titres and titre-rises against A(H3N2) viruses spanning 1968 to 2018 by prior vaccination status. (A-B)** Year 1 (2017/18) day 0 geometric mean titres (GMT) and Geometric mean rises (GMR) in titres between day 0 and day 30. **(C-D)** Year 2 (2018/19) Day 0 GMTs and Day 30 GMRs. Asterisks indicate p values for Wilcox test comparing 0/5 versus 5/5 prior vaccination groups: * < 0.05, ** < 0.01, *** < 0.001. Vaccines strains are shaded in yellow.


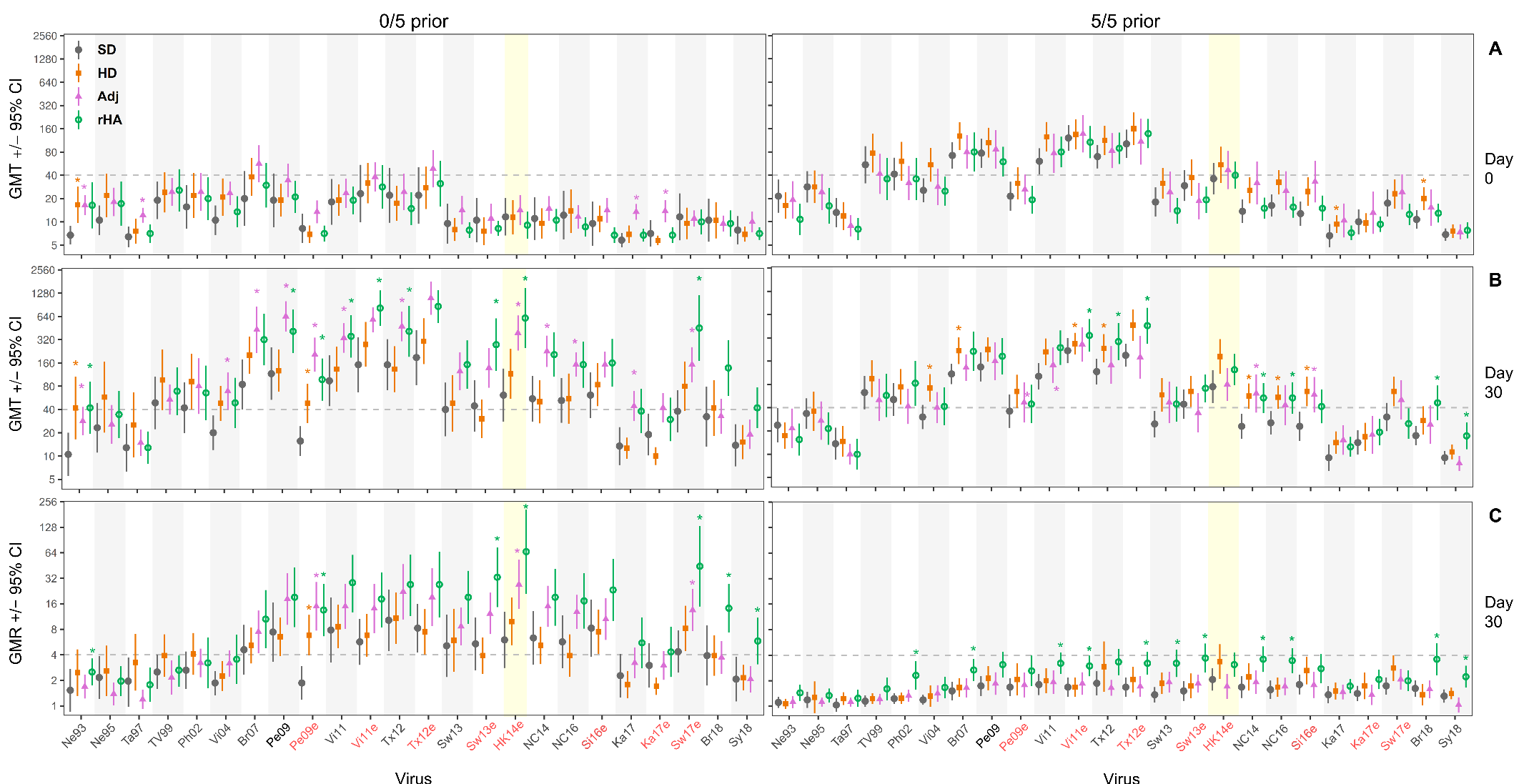


**Figure S3. Year 1 HAI antibody titres and titre-rises against A(H3N2) viruses spanning 1993 to 2018 by vaccine received and prior vaccination status.** **(A)** day 0 geometric mean titres (GMT). **(B)** day 30 GMTs. **(C)** Geometric mean rise (GMR) in titres between day 0 and day 30. Asterisks indicate p values < 0.05 for the comparison of GMTs or GMRs for each virus between SD and enhanced (HD, Adj, rHA) vaccines by Wilcox test. Egg-grown viruses are indicated by red x-axis labels. The 2017/18 vaccine strain is shaded yellow.


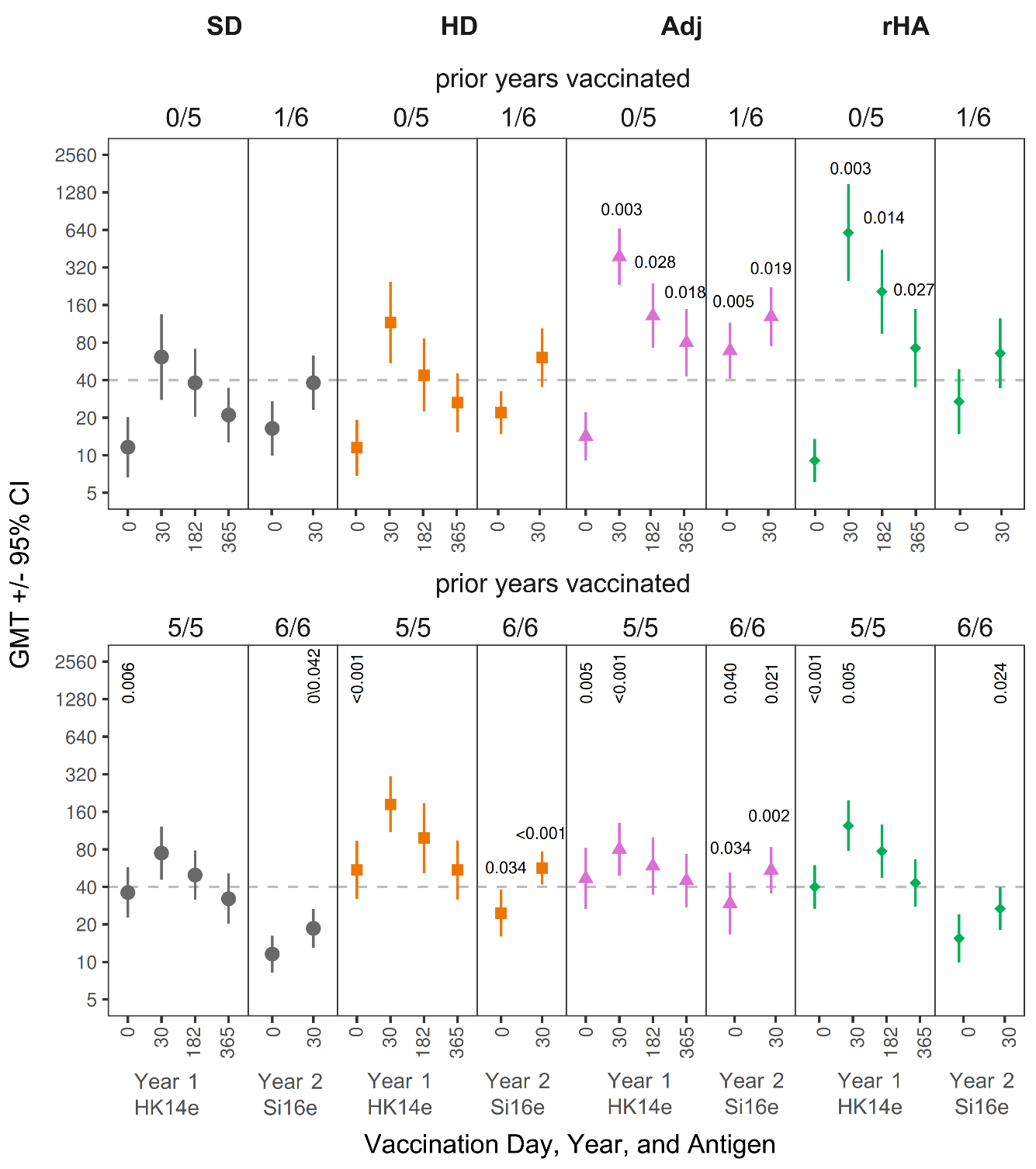
 **Figure S4. Time course of HAI antibody titres against A(H3N2) vaccine strains by type of vaccine received and vaccination history.** Geometric mean titres (GMTs) with 95% confidence intervals (CI) against egg-grown A/Hong Kong/4801/2014 (HK14e) in year 1 and A/Singapore/INFIMH-16-0019/2016 (Si16e) in year 2. Wilcoxon ranked sum p values for comparison of enhanced versus SD vaccine (horizontal text) or of 0/5 versus 5/5 prior vaccinations (vertical text, bottom panel only) are shown if p < 0.05.


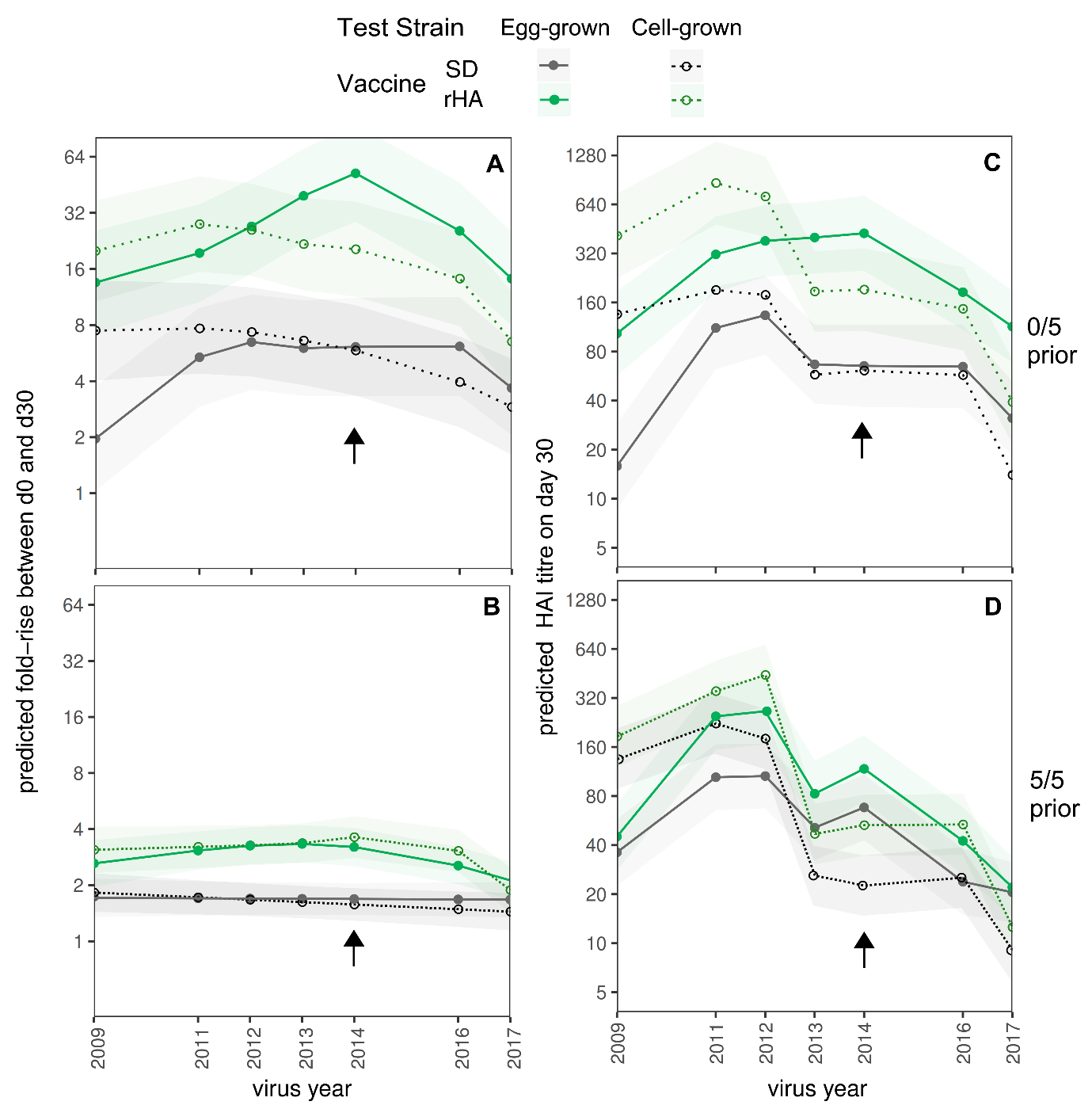


**Figure S5. HAI antibody responses against egg- versus cell-grown equivalents of viruses circulating between 2009 and 2017.** Generalized additive models were used to estimate titres and titre rises for egg-grown and cell-grown viruses grouped by year of circulation. Results are shown for year 1 for participants who received SD or rHA vaccine (legend) and who had been vaccinated 0/5 versus 5/5 prior years. Arrows indicate the vaccine strain year.


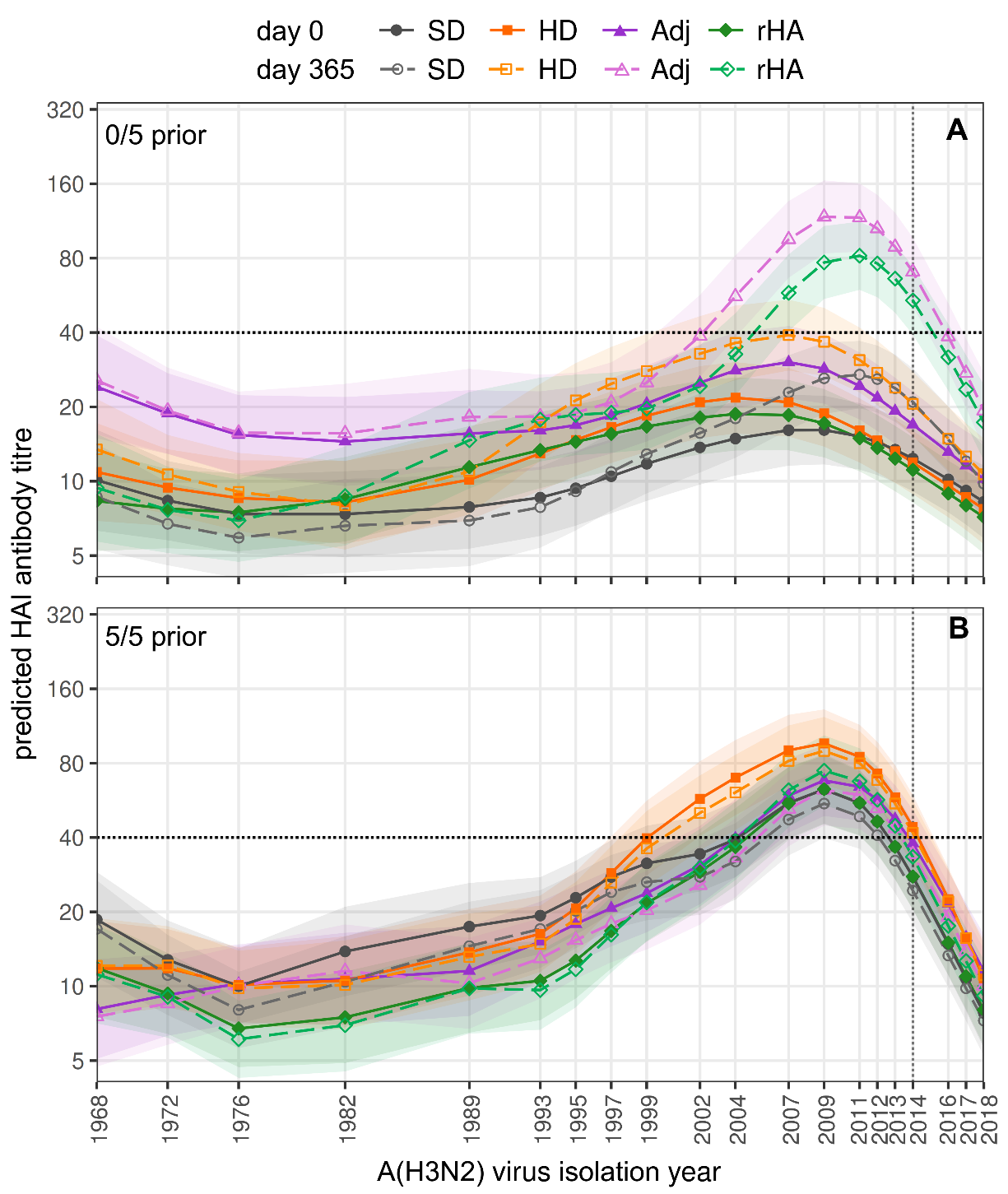


**Figure S6. HAI antibody landscapes before versus 1 year after vaccination.** Generalized additive models were used to estimate titres for viruses grouped by year of circulation. Results are shown for participants vaccinated 0/5 prior years **(A)** and 5/5 prior years **(B)** who received SD, HD, Adj, or rHA vaccine (legend in panel A). Solid lines and filled symbols represent titre landscapes on day 0 of year 1 and dashed lines and open symbols represent landscapes on day 365 (~ d0 year 2).
